# Knowledge mapping of acupoint sensitization and acupoint specificity: a bibliometric analysis

**DOI:** 10.3389/fnins.2023.1292478

**Published:** 2023-11-20

**Authors:** Xuesong Wang, Xuxin Li, Yuanbo Gao, Di Wang, Jun Liu, Xisheng Fan, Hao Chen, Guang Zuo, Haiping Li, Xiaojun Zheng, Xifen Zhang, Juncha Zhang, Yanfen She

**Affiliations:** ^1^College of Acupuncture-Moxibustion and Massage, Hebei University of Chinese Medicine, Shijiazhuang, Hebei, China; ^2^Hebei International Joint Research Center for Dominant Diseases in Chinese Medicine and Acupuncture, Hebei University of Chinese Medicine, Shijiazhuang, Hebei, China

**Keywords:** bibliometrics, acupoint sensitization, acupoint specificity, CiteSpace, VOSviewer, R software

## Abstract

**Objective:**

The relationship between acupoint sensitization and acupoint specificity is a topic of significant interest in acupuncture research. Numerous clinical studies have demonstrated that needling pain sensitive acupoints yields superior therapeutic outcomes compared to traditional acupoints, particularly in the context of pain disorders. However, there is a lack of bibliometric analysis in acupuncture area. Therefore, the objective of this study is to offer a comprehensive overview of the knowledge structure and research hotspots pertaining to acupoint sensitization and acupoint specificity.

**Methods:**

The search for publications pertaining to acupoint sensitization and acupoint specificity was conducted in the Web of Science Core Collection (WoSCC) database from its inception until August 11, 2023. Subsequently, bibliometric analyses were carried out using VOSviewer, CiteSpace, R software (Bibliometrix package), and GraphPad Prism software.

**Results:**

This study includes 4,940 articles from 72 countries, with China and the United States being the leading countries. The number of publications related to acupoint sensitization and specificity has been increasing annually. Major research institutions involved in this field include the Shanghai University of Traditional Chinese Medicine, Kyung Hee University, Beijing University of Chinese Medicine, Chinese Academy of Chinese Medical Sciences, and China Medical University, among others. “Evidence-based Complementary and Alternative Medicine” is the most popular journal in acupuncture field, and “PAIN” is the most co-cited journal. Publications are contributed by 20,325 authors from all over the world, with Wu Huangan, Fang Jianqiao, Lin Yi-Wen, Liu Huirong, and Chen Rixin having published the most articles. Han Ji-Sheng is the most cited author in this research area. The main directions include the study of temperature specificity of acupoints, the diagnosis of acupoint sensitization diseases, and the study of the mechanism of acupoint sensitization. The most listed keywords in recent years are “TRPV1,” “signaling pathway,” and “diagnosis.”

**Conclusion:**

This is the first bibliometric study to comprehensively summarize research trends and advances in acupoint sensitization and acupoint specificity, and the information highlights recent research preliminary and main directions that serve as a reference for acupoint sensitization and acupoint specificity research.

## 1 Background

According to traditional acupuncture and the moxibustion theory, meridians serve as pathways to connect with the *Zang-Fu (脏腑)*, communicate with the internal and external, run the *Qi (气)* and blood, nutrients, defend against disease, respond to disease, conduction and induction, and balance the role of *Yin (阴)* and *Yang (阳)*. Acupoints are a special area of the body where the *Qi (气)* of the *Zang-Fu (脏腑)* and meridians converge on the surface of the body, and they are important components of the meridian composition. On the one hand, stimulation of the relevant acupoints can regulate the function of *Zang-Fu (脏腑)*, which is the key to the therapeutic role of acupuncture. On the other hand, the acupoints are a special area to respond to the function of the *Zang-Fu (脏腑)* and play an important role in assisting disease diagnosis.

There have been records of “*response point (应动点)*” in *Miraculous Pivot (《灵枢》)*, and the ancients used “response point” to locate acupoints. The “response point” is the local pathological reaction of acupoints in the illness status, which is now commonly referred to as the “sensitization point” (Tan et al., 2019). Because of the different functional conditions of their target organs, the function of acupoints reacting to diseases will change. Sensitization phenomena such as abnormal pain sensation will be experienced in specific locations of the body surface when the body is in a diseased state (Zhu, 2015); With the healing of the disease, the sensitization phenomena will gradually diminish or even disappear (Chen et al., 2011). Acupoint sensitization is a mechanism that allows little stimuli to have great effects, and it is a critical fulcrum for enhancing therapeutic efficacy (Huang et al., 2021). Currently, pain sensitization (Liu et al., 2023), heat sensitization (Li et al., 2016; Xie et al., 2016), microcirculation sensitization (Ding et al., 2018), electrical sensitization (Reichmanis et al., 1975; Ahn and Martinsen, 2007), acoustic sensitization (Wei and Tu, 2005), and light sensitization (Shen et al., 2008) are common forms of acupoint sensitization, with pain sensitization and heat sensitization being the most common manifestations of acupoint sensitization (Zhu, 2019).

Under physiological conditions, acupoint areas exhibit some specificity compared to non-acupoint areas. For example, the method of determining skin electrical resistance was utilized to reveal that Ryodoraku is comparable to the meridians, i.e., it uses electrical characteristics (Lee et al., 2005). Similarly, our team employed infrared thermography to determine skin temperature at the relevant acupoints and some differences in skin temperature, i.e., thermal characteristics were discovered (Fan et al., 2022; Wang et al., 2023). Furthermore, the meridians have optical characteristics (Yang et al., 2007) and sound characteristics (Xiong et al., 2023). Both acupoint sensitization and acupoint specificity are essential generalizations in the field of acupuncture in Chinese medicine. They share a link, although there are some differences (Zhu, 2021). Acupoint specificity is a fundamental concept that emphasizes that each acupoint has unique biophysical properties. Acupoint sensitization refers to a change in sensitivity that includes biophysical properties when the body is in a pathological state in which certain acupoints become more sensitive. Furthermore, acupoint sensitization does not alter the specificity of an acupoint, but rather improves its therapeutic impact.

Bibliometrics is a method of literature analysis that allows for quantitative and qualitative analysis of detailed information about authors, keywords, journals, countries, institutions, references, etc., in a relevant field of study, and can be used to analyze the publications’ output in a specific field of study from multiple perspectives (Wang et al., 2019; Ke et al., 2020). Commonly used bibliometric tools such as CiteSpace (Synnestvedt et al., 2005), VOSviewer (Yeung et al., 2020), the R package “Bibliometrix” (Li et al., 2020), and HistCite (Lu et al., 2020) can be used to visualize and analyze the results, and in recent years, have been widely used in the field of acupuncture and moxibustion research, such as lumbar pain (Jiao et al., 2022), myofascial syndromes (Luo et al., 2023), shoulder pain (Xu et al., 2023), vomiting (Tan et al., 2023), neck pain (Gong et al., 2023), and facial paralysis (Yu et al., 2023). These studies have focused on the assessment of the clinical efficacy of acupuncture, however, bibliometric studies on the essence of acupoint sensitization and acupoint specificity have not yet been reported. Therefore, this study aimed to conduct bibliometric analysis of published publications on acupoint sensitization and acupoint specificity to identify the main contributors and the current state of research, as well as to identify research trends and future prospects in acupuncture research.

## 2 Methods

### 2.1 Search strategy

Web of Science Core Collection (WoSCC) was used to search the related articles to acupoint sensitization and acupoint specificity. The search was performed from the date of the establishment of this database to August 11, 2022, using a combination of medical terms and words. English search terms included Acupuncture, Acupoints, Acupoint, Needle Therapy, Needling, meridian, Acupoint sensitization, Moxibustion, Moxibustion therapy, Pain, diagnosis, physiopathology, infrared, thermal, temperature, skin temperature, heat sensitization, heat-sensitization, Central Nervous System Sensitization, Neurogenic Inflammation, Pain Measurement, Pain Threshold, hyperalgesia, algesia, electric, Conductivity, Electrophysiology, electr* impedance, Acoustics, acoustic propert*, sound wave*, light propert*, radiation spectrum, receptive field, and morphology. Literature types were restricted to “Article” and “Review,” and the full search strategy is shown in Table 1.

**Table 1:** Detailed search strategy for Web of Science Core Collection (WoSCC).

No.	Search item
#1	TS = (“Acupuncture* “OR “Acupoints” OR “Acupoint” OR “acupoint*” OR “Electroacupuncture*” OR “Electro-acupuncture” OR “Acupoint Therapy” OR “Acupuncture Treatment” OR “Acupuncture Treatments” OR “Needle Therapy” OR “Needling” OR “meridian” OR “Meridians” OR “needling” OR “Acupoint sensitization” OR “Moxibustion” OR “Moxibustion therapy”) OR TI = (“Acupuncture* “OR “Acupoints” OR “Acupoint” OR “acupoint*” OR “Electroacupuncture*” OR “Electro-acupuncture” OR “Acupoint Therapy” OR “Acupuncture Treatment” OR “Acupuncture Treatments” OR “Needle Therapy” OR “Needling” OR “meridian” OR “Meridians” OR “needling” OR “Acupoint sensitization” OR “Moxibustion” OR “Moxibustion therapy”) OR AB = (“Acupuncture* “OR “Acupoints” OR “Acupoint” OR “acupoint*” OR “Electroacupuncture*” OR “Electro-acupuncture” OR “Acupoint Therapy” OR “Acupuncture Treatment” OR “Acupuncture Treatments” OR “Needle Therapy” OR “Needling” OR “meridian” OR “Meridians” OR “needling” OR “Acupoint sensitization” OR “Moxibustion” OR “Moxibustion therapy”)
#2	TS = ((“Pain” AND “diagnosis”) OR (“Pain” AND “physiopathology”)) OR TI = ((“Pain” AND “diagnosis”) OR (“Pain” AND “physiopathology”)) OR AB = ((“Pain” AND “diagnosis”) OR (“Pain” AND “physiopathology”))
#3	TS = (“infrared” OR “thermal” OR “temperature” OR “skin temperature” OR “Skin Temperatures” OR “heat sensitization” OR “heat-sensitization”) OR TI = (“infrared” OR “thermal” OR “temperature” OR “skin temperature” OR “Skin Temperatures” OR “heat sensitization” OR “heat-sensitization”) OR AB = (“infrared” OR “thermal” OR “temperature” OR “skin temperature” OR “Skin Temperatures” OR “heat sensitization” OR “heat-sensitization”)
#4	TS = (“Central Nervous System Sensitization” OR “Neurogenic Inflammation” OR “sensiti*” OR “Pain Measurement” OR “Pain Threshold” OR “hyperalgesia” OR “algesia”) OR TI = (“Central Nervous System Sensitization” OR “Neurogenic Inflammation” OR “sensiti*” OR “Pain Measurement” OR “Pain Threshold” OR “hyperalgesia” OR “algesia”) OR AB = (“Central Nervous System Sensitization” OR “Neurogenic Inflammation” OR “sensiti*” OR “Pain Measurement” OR “Pain Threshold” OR “hyperalgesia” OR “algesia”)
#5	TS = (“lectric Conductivity” OR “Electrophysiology” OR “electr* impedance” OR “Acoustics” OR “acoustic propert*” OR “sound wave*” OR “light propert*” OR “radiation spectrum “OR “receptive field” OR “morphology”) OR TI = (“lectric Conductivity” OR “Electrophysiology” OR “electr* impedance” OR “Acoustics” OR “acoustic propert*” OR “sound wave*” OR “light propert*” OR “radiation spectrum “OR “receptive field” OR “morphology”) OR AB = (“lectric Conductivity” OR “Electrophysiology” OR “electr* impedance” OR “Acoustics” OR “acoustic propert*” OR “sound wave*” OR “light propert*” OR “radiation spectrum “OR “receptive field” OR “morphology”)
#6	#2 OR #3 OR #4 OR #5
#7	#1 AND #6

### 2.2 Data analysis

GraphPad Prism software (version 9.0.3), VOSviewer (version 1.6.18), CiteSpace (version 6.1.R1), and R software (Bibliometrix package, version 3.2.1) were used to perform quantitative and qualitative analyses of the literature obtained from the database retrieval. First, GraphPad Prism software was used to quantitatively analyze the number of publications per year. Then, VOSviewer software was used to analyze countries and institutions, journals and co-cited journals, and authors and co-cited authors. In the co-occurring network, in graphs generated by VOSviewer, a node represents an item such as a country, institution, journal, or author (Arruda et al., 2022; Shen et al., 2022). For the partitioning and impact factor of the journals, we referred to the Journal Citation Report 2022. Next, CiteSpace software was used to create a dual-map overlay of journals used to show citation relationships between journals and co-cited journals, and to identify references with strong citation bursts (Chen et al., 2012). Finally, R software (Bibliometrix package)^1^ was used to analyze trending topics of the keywords and to construct the overall contribution network of the topic terms related to acupoint sensitization and acupoint specificity (Aria and Cuccurullo, 2017).

## 3 Results

### 3.1 Quantitative analysis of publication

**Figure 1:**
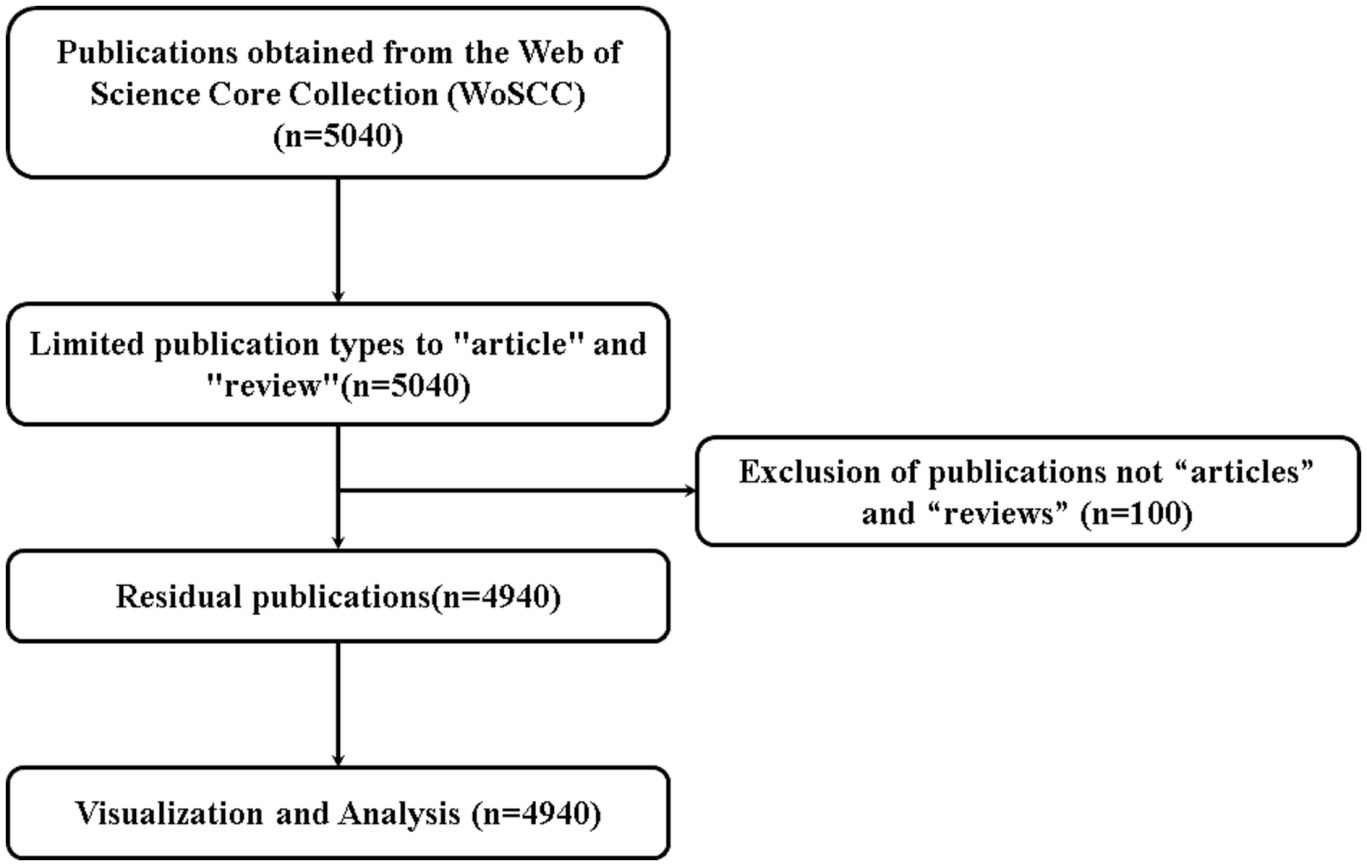
Publications screening flowchart.

**Figure 2:**
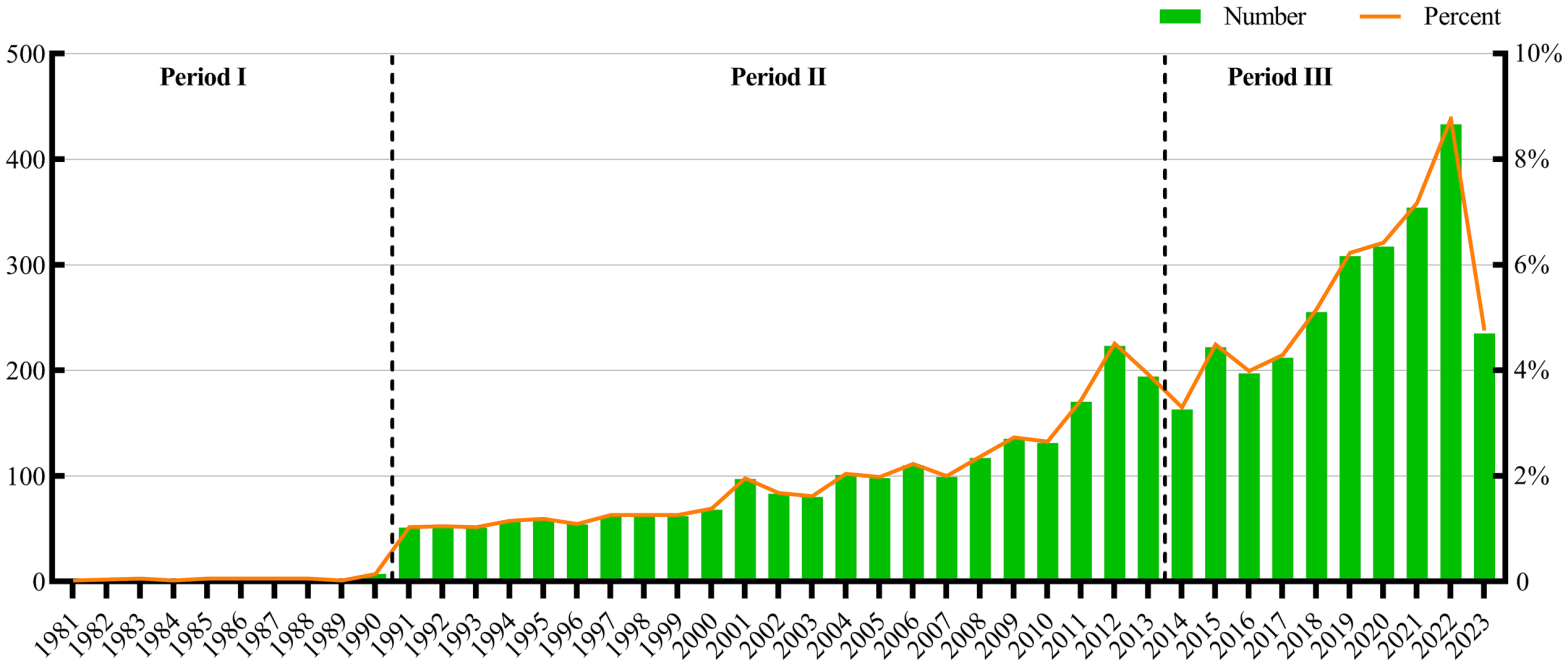
Annual research results on acupoint sensitization and acupoint specificity.

In this study, a total of 4,940 related publications were retrieved, the earliest of which was reported in 1981, including 4,391 articles and 549 reviews (Figure 1). The number of publications increased annually, and according to the number of publications per year, publications can be categorized into three phases: the first phase (1981–1990), the beginning of the research, in which the number of publications per year was less than 10. In the second phase (1991–2010), in which the overall number of annual publications increased annually, the number of publications per year was less than 135, in the range of 51–135, and in the third phase (2011–2022), the number of publications per year began to increase dramatically, and the average number of publications per year was 254. The number of publications in 2022 was 2.5 times the number of publications in 2011. The growth rate of the number of publications in the third phase was significantly higher than that in the first two phases (Figure 2). Since the deadline for the search strategy was August 11, 2023, the number of publications in 2023 will decrease, and it is predicted that the number of publications in 2023 will be at least the same as that in 2022, or even exceed the number in 2022.

### 3.2 Country and institutional analysis

**Figure 3:**
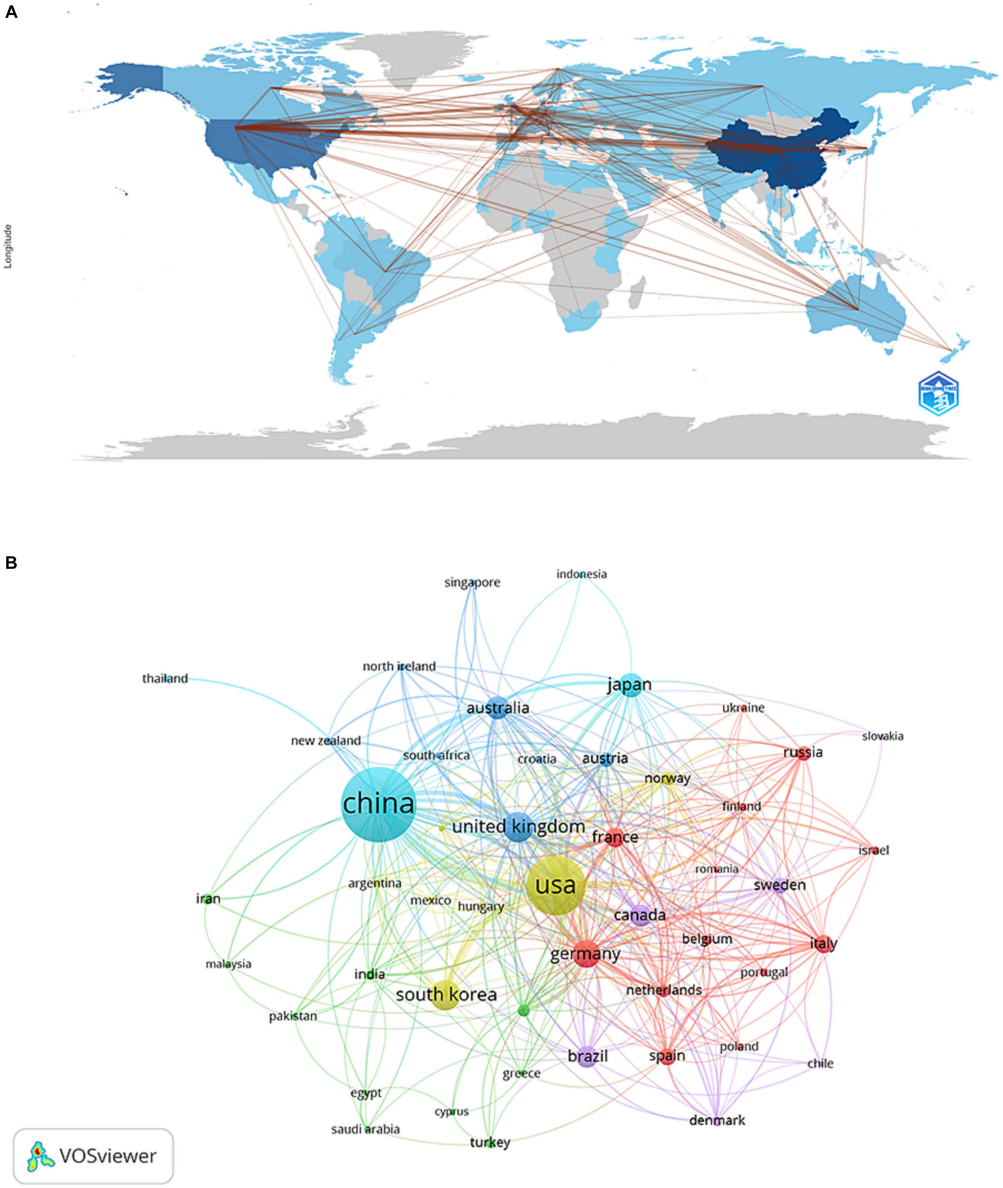
The geographical distribution **(A)** and visualization of countries **(B)** on acupoint sensitization and acupoint specificity (**A**: the color shades represent the number of published publications; **B**: different nodes represent different countries, and the size of the nodes represents the number of publications).

The publications selected originated from 72 countries and 3,509 institutions. The top ten countries were distributed among China, USA, Korea, Germany, United Kingdom, Japan, Brazil, Australia, Canada, and France, of which the countries with the most publications were China (*n* = 1755, 35.53%) and the United States (*n* = 890, 18.02%) (Table 2). Moreover, China and the United States accounted for almost half of the total number of publications (Table 2). Moreover, countries with the number of publications greater than or equal to five were filtered and a visualization network was created (Figure 3). The data showed that there was more active cooperation between different countries, such as China, USA, and the United Kingdom.

**Table 2:** Top 10 countries and institutions on research of acupoint sensitization and acupoint specificity.

**Country**	**Articles**	**Affiliation**	**Articles**
China	1755	Shanghai University of Traditional Chinese Medicine	1,151
USA	890	Kyung Hee University	119
Korea	285	Beijing University of Chinese Medicine	117
Germany	212	Chinese Academy of Chinese Medical Sciences	115
United Kingdom	185	China Medical University	107
Japan	175	Fudan University	97
Brazil	151	Chinese Academy of Sciences	96
Australia	141	Chengdu University of Traditional Chinese Medicine	94
Canada	106	Zhejiang Chinese Medicine University	73
France	88	Guangzhou University of Chinese Medicine	70

**Figure 4:**
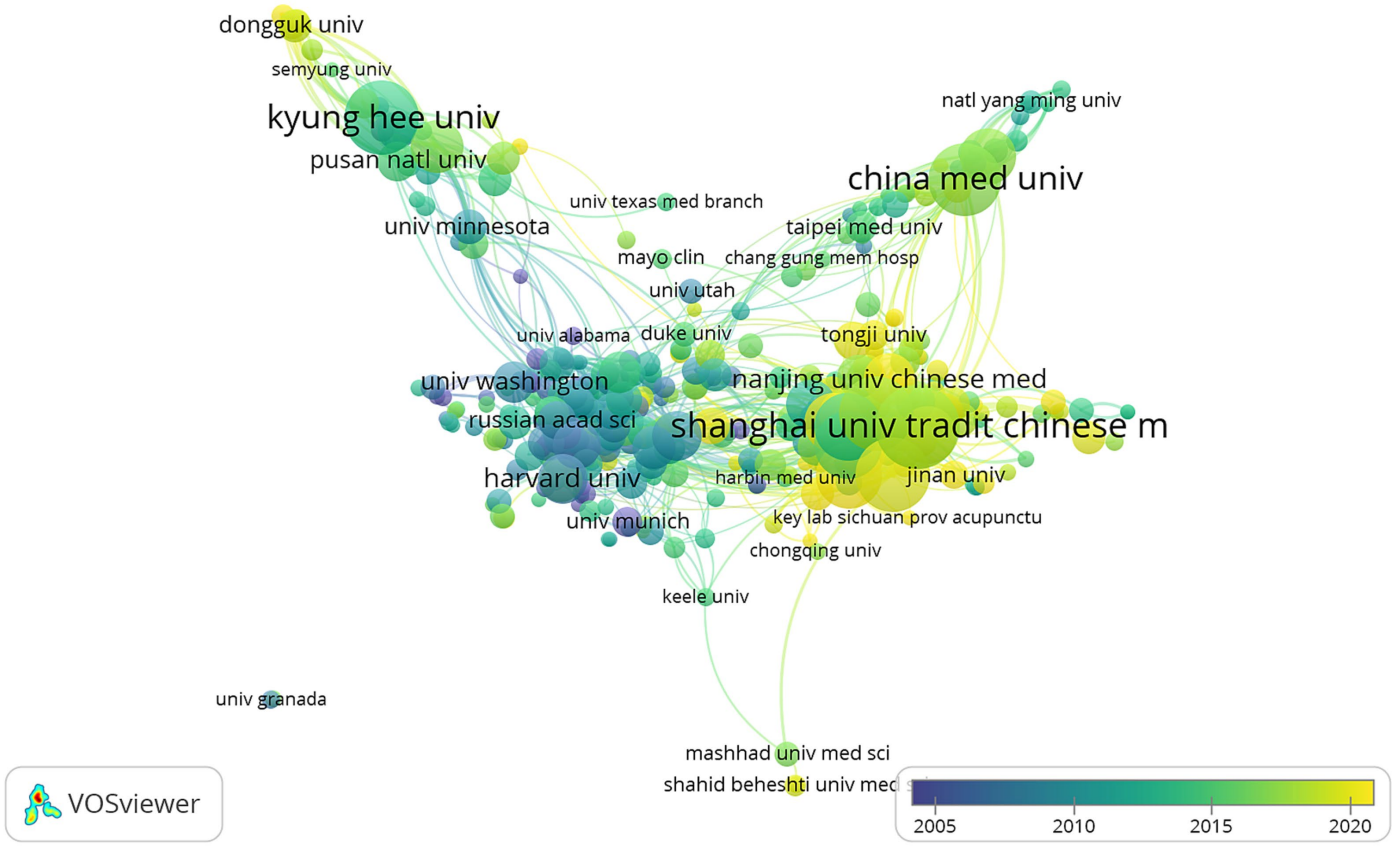
The visualization of institution on acupoint sensitization and acupoint specificity (different nodes represent different institutions, the size of the node represents the number of publications; the color of the node represents different years).

The top 10 institutions in publications were found in four countries, with six of them being in China. The top 10 institutions included the Shanghai University of Traditional Chinese Medicine, Kyung Hee University, Beijing University of Chinese Medicine, Chinese Academy of Chinese Medical Sciences, China Medical University, Fudan University, Chinese Academy of Sciences, Chengdu University of Traditional Chinese Medicine, Zhejiang Chinese Medicine University, and the Guangzhou University of Chinese Medicine (Table 2). The network was visualized with a minimum number of publications of 5 (Figure 4). The Shanghai University of Traditional Chinese Medicine, Beijing University of Chinese Medicine, Chengdu University of Traditional Chinese Medicine, and the Chinese Academy of Chinese Medical Sciences closely collaborate. In addition, UNIV GRANADA also had a high number of publications but did not collaborate with other institutions.

### 3.3 Journals and co-cited journals

**Figure 5:**
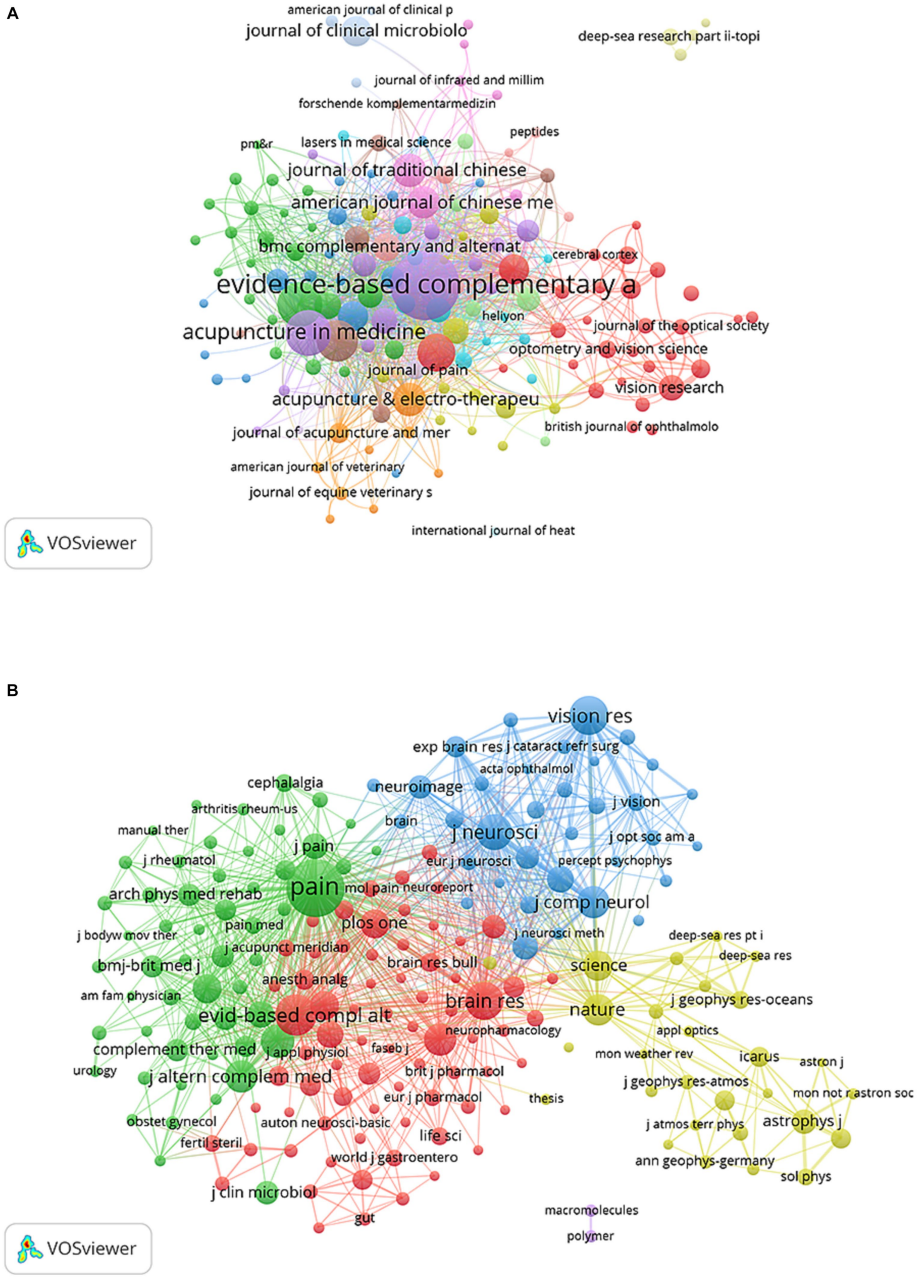
The visualization of journals **(A)** and co-cited journals **(B)** on acupoint sensitization and acupoint specificity (Different nodes represent different journals; the size of the node represents the number of publications; different colors represent different clusters).

Publications related to the study of acupoint sensitization and acupoint specificity were published in a total of 1,456 journals. Evidence-based Complementary and Alternative Medicine had the highest number of publications (*n* = 272, 5.51%), followed by Acupuncture in Medicine (*n* = 118, 2.39%), Medicine (*n* = 114, 2.30%), Journal of Alternative and Complementary Medicine (*n* = 100, 2.02%), and the Journal of Acupuncture and Tuina Science (*n* = 81, 1.64%). Among the top 15 journals with the most engaged publications, with a reference to Journal Citation Report 2022, journals with the highest impact factor included Neural Regeneration Research (IF = 6.1), American Journal of Chinese Medicine (IF = 5.7), and Frontiers in Neuroscience (*n* = 4.3) (Table 3). Subsequently, the 175 journals with several publications greater than or equal to five were visualized (Figure 5A). Evidence-based Complementary and Alternative Medicine was compared with Acupuncture in Medicine, Medicine, and Acupuncture Electro-Therapeutics Research and had an active citation relationship.

**Figure 6:**
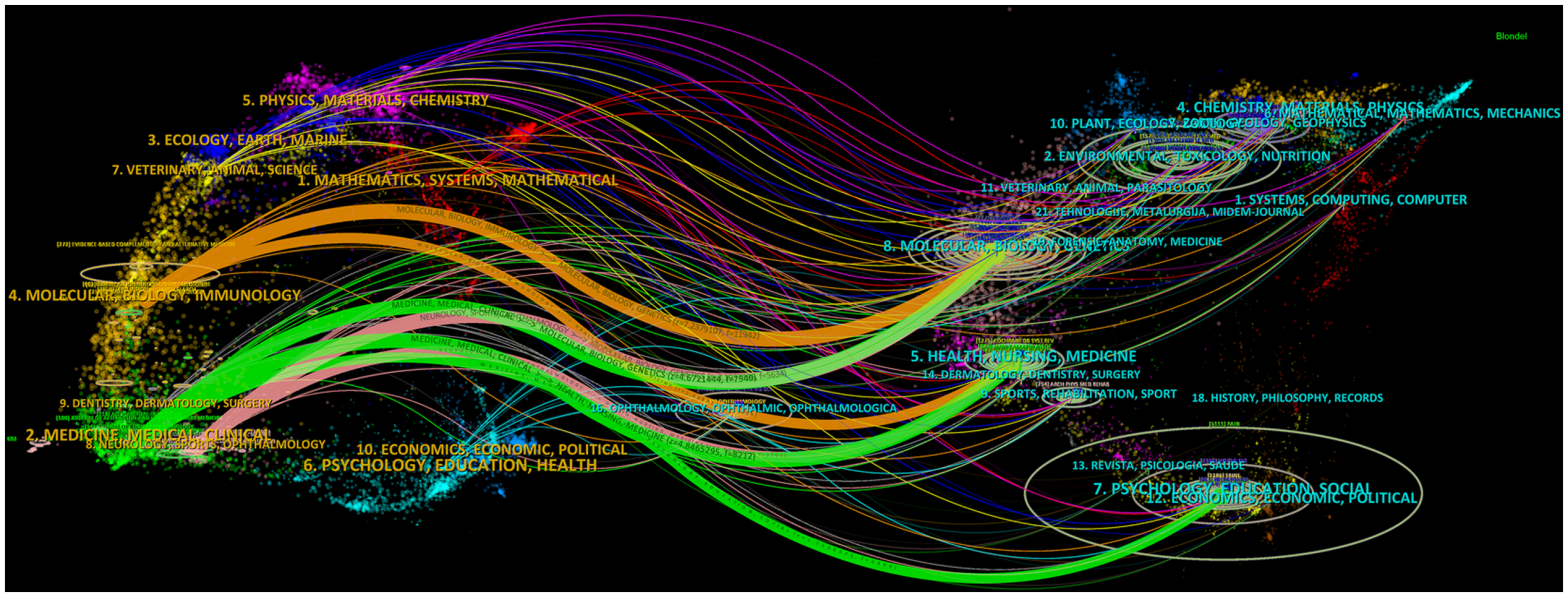
The dual-map overlay of journals on research of acupoint sensitization and acupoint specificity (the left side represents the citing journal clusters; the right side represents the cited journal clusters; different lines represent different citation routes).

Co-cited journals mean that two or more journals are co-cited in the same publication. There is a co-citation relationship between two or more journals, and the co-cited times are the co-citation strength. The higher the co-citation strength, the closer and more relevant the relationship between the two journals (Hood and Wilson, 2001). Among the top 10 co-cited journals, five journals were cited more than 2,000 times, and the most cited journal was PAIN (co-citation = 5,120), followed by Evidence-based Complementary and Alternative Medicine (co-citation = 2,611), Journal of Geophysical Research-Space Physics (total citations = 2,493), Vision Research (total citations = 2,333), and Brain Research (total citations = 2,280) (Table 3). In addition, the impact factor of the journal Nature was the highest (IF = 64.8), followed by Science (IF = 56.9), and the Cochrane Database of Systematic Reviews (IF = 8.4). A total of 196 journals with a minimum number of co-citations equal to 150 were filtered to map the co-citation network (Figure 5B). PAIN with Evidence-based Complementary and Alternative Medicine, Brain Research, Acupuncture in Medicine, Journal of Alternative and Complementary Medicine, and others had positive co-citation relationships. The dual-map overlay of journals shows the citation relationships between journals and co-cited journals, with clusters of citing journals on the left and clusters of cited journals on the right (Chen, 2017). As shown in Figure 6, the orange, green, and pink paths are the major citation paths, which indicate that studies published in Molecular/Biology/Immunology/Medicine/Medical/Clinical/Neurology journals are mainly cited by Molecular/Biology/Genetics/ Health/Medicine journals.

**Table 3:** Top 15 journals and co-cited journals for research on acupoint sensitization and acupoint specificity.

Rank	Journal	Count	IF	*Q*	Co-cited Journal	Co-citation	IF	*Q*
1	Evidence-based Complementary and Alternative Medicine	272	–	–	Pain	5,120	7.4	1
2	Acupuncture in Medicine	118	2.5	3	Evidence-based Complementary and Alternative Medicine	2,611	–	–
3	Medicine	114	1.6	3	Vision Research	2,333	3.6	2
4	Journal of Alternative and Complementary Medicine	100	2.6	3	Brain Research	2,280	2.9	3
5	Journal of Acupuncture and Tuina Science	81	0.5	–	Neuroscience	1941	3.3	3
6	Journal of Traditional Chinese Medicine	66	2.6	3	Acupuncture in Medicine	1721	2.5	3
7	Acupuncture Electro-Therapeutics Research	66	0.3	4	Neuroscience Letters	1,580	2.5	3
8	American Journal of Chinese Medicine	62	5.7	1	Journal of Alternative and Complementary Medicine	1,579	2.6	3
9	Plos One	53	3.7	2	Journal of Comparative Neurology	1,578	2.5	1
10	Journal of Pain Research	52	2.7	3	Nature	1,546	64.8	1
11	Bmc Complementary and Alternative Medicine- > BMC Complementary Medicine And Therapies	44	3.9	2	Investigative Ophthalmology and Visual Science	1,495	4.4	1
12	Trials	41	2.5	5	Science	1,408	56.9	1
13	Frontiers in Neuroscience	39	4.3	2	Plos One	1,314	3.7	2
14	Neural Regeneration Research	38	6.1	1	American Journal Of Chinese Medicine	1,310	5.7	1
15	Complementary Therapies in Medicine	35	3.6	2	Cochrane Database Of Systematic Reviews	1,279	8.4	1

### 3.4 Authors and co-cited authors

**Figure 7:**
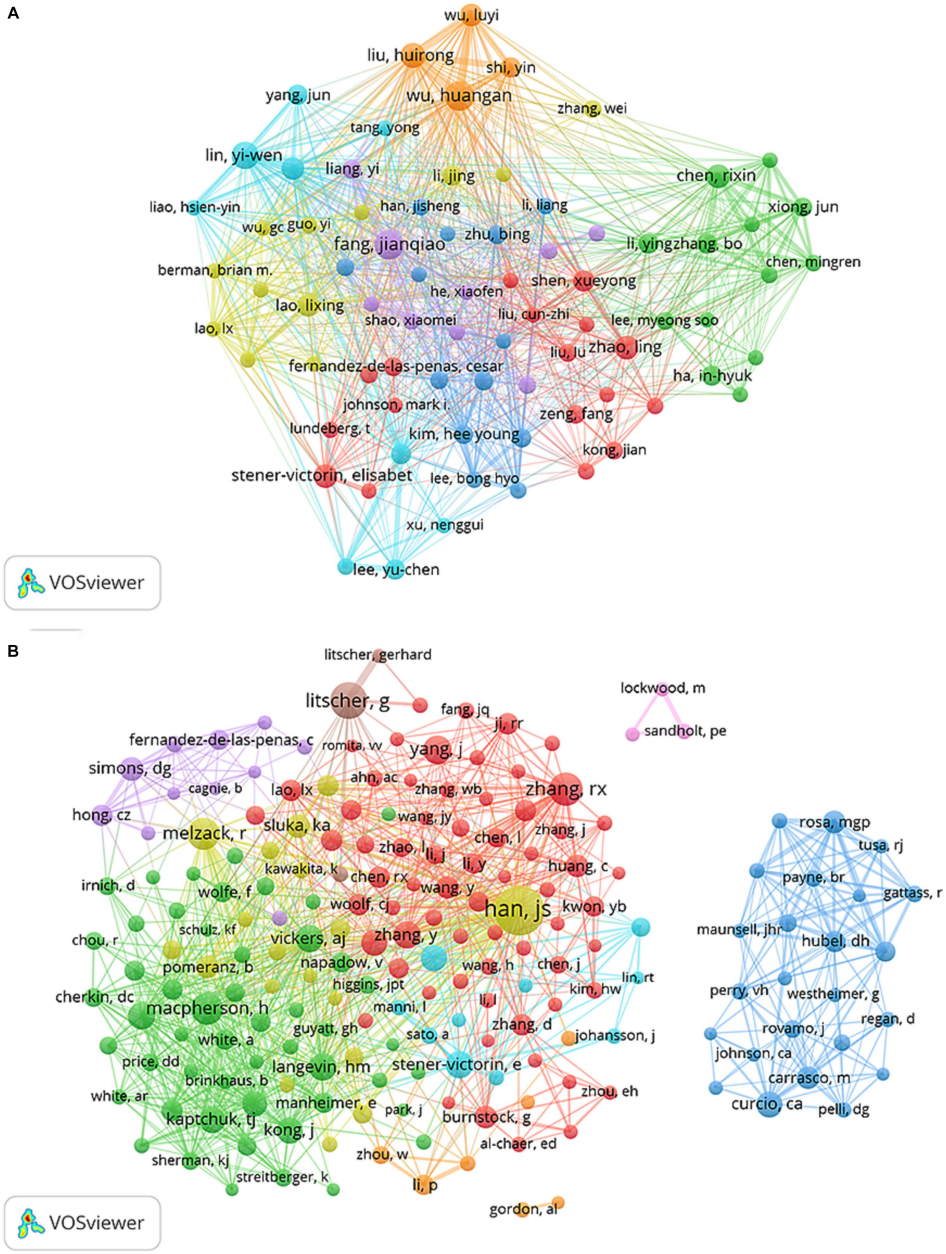
The visualization of authors **(A)** and co-cited authors **(B)** on research of acupoint sensitization and acupoint specificity (Different nodes represent different authors; the size of the node represents the number of publications; different colors represent different clusters).

A total of 20,325 authors were involved in acupoint sensitization and acupoint specificity studies. Sorting the authors according to the number of publications, among the top 20 authors, the lowest number of publications was 17 (Table 4). A collaborative network was constructed for the 79 authors with more than or equal to 10 publications (Figure 7A), and it was found that Wu Huangan, Fang Jianqiao, Lin Yi-Wen, Liu Huirong, and Chen Rixin had the largest nodes because they published the most relevant publications. In addition, the collaboration between multiple authors was closely observed. For example, Wu Huangan, Fang Jianqiao, Lin Yiwen, Fang Jianqiao, Liu Cunzhi, Zhaoling, and Shen Xueyong closely collaborated. Fang Jianqiao, Han Ji, Li Man, Liang Yi, and Zhu Bin closely collaborated, and Chen Rixin, Lin Yi-Wen, Yang Jun, Liu Huirong, Li Ying, and Zhang Bo closely collaborated.

Co-cited authors refer to two or more authors who are co-cited in the same publication, there is a co-citation relationship between these two or more authors, and the co-cited times are the co-citation strength. The higher the co-citation strength, the closer the relationship between these authors and the higher the relevance (Hood and Wilson, 2001). Among all co-cited authors, 11 authors were co-cited more than 200 times (Table 4). The author with the highest number of co-citations was Han JS (*n* = 674), followed by Litscher G (*n* = 364) and Zhang RX (*n* = 303). In addition, the 193 authors with co-citation counts greater than 50 were filtered for mapping the co-citation network (Figure 7B). Active collaborations were also observed between different co-cited authors, such as Han JS and Zhang Y, Macpherson H, and Simons DG; Curcio Ca and Hubel DH, etc.

**Table 4:** Top 10 authors and co-cited authors on research of acupoint sensitization and acupoint specificity.

Rank	Authors	Count	Co-cited authors	Citations
1	Wu Huangan	39	Han JS	647
2	Fang Jianqiao	37	Litscher G	364
3	Lin Yi-Wen	33	Zhang RX	303
4	Liu Huirong	27	Melzack R	280
5	Chen Rixin	25	Macpherson H	273
6	Stener-Victorin Elisabet	25	Yang J	224
7	Zhao Ling	24	Langevin Hm	214
8	Hsieh Ching-Liang	23	Stener-Victorin E	206
9	Lao Lixing	23	Zhang Y	205
10	Wu Luyi	22	Ernst E	203

### 3.5 Co-cited references

**Figure 8:**
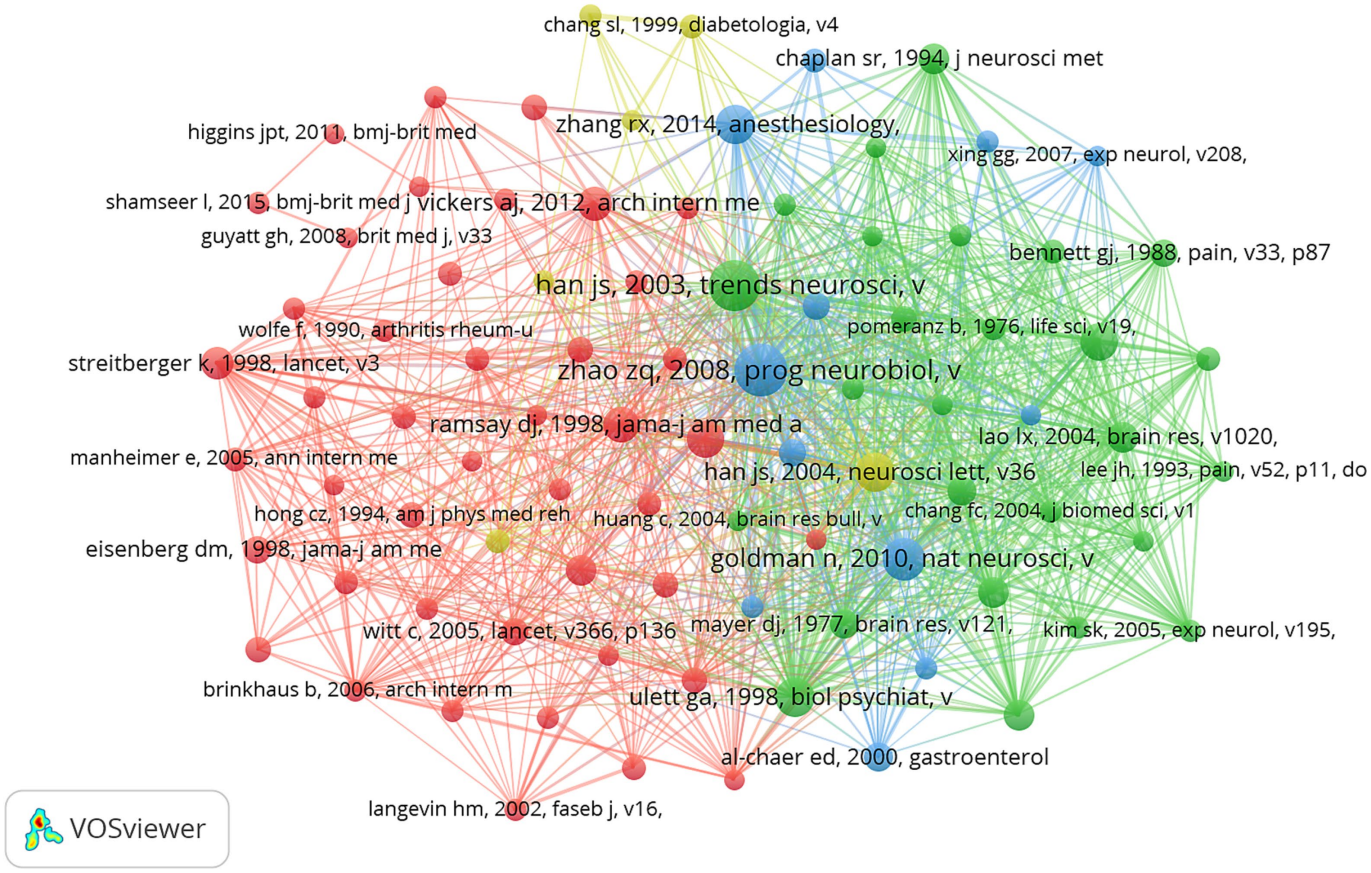
The visualization of co-cited references on research of acupoint sensitization and acupoint specificity (Different nodes represent different references; the size of the node represents the number of publications; different colors represent different clusters).

Co-cited references mean that two or more references are co-cited in the same publication, there is a co-citation relationship between these two or more references, and the co-cited times are the co-citation strength. The higher the co-citation strength, the closer the relationship between these references and the higher the relevance (Hood and Wilson, 2001). Over the past 20 years, 25 of the co-cited literature off on acupoint sensitization and acupoint specificity all had a co-citation frequency greater than 50. Among the top 10 co-cited references (Table 5), all references were co-cited at least 82 times, with 8 references co-cited more than 100 times. Documents with a citation frequency greater than or equal to 30 were selected to construct the co-citation network map (Figure 8). As shown in Figure 8, “Zhao ZQ, 2008, Prog Neurobiol” and “Han JS, 2003, Trends Neurosciense,” “Han JS, 2003, Neurosciense Letters” and “Zhang RX, 2014, Anesthesiology” were more likely to be cited simultaneously.

**Table 5:** Top 10 co-cited references on research of acupoint sensitization and acupoint specificity.

Rank	Cited references	Citations
1	Zhao ZQ, 2008, Prog Neurobiol, V85, P355, DOI 10.1016/J.PNEUROBIO.2008.05.004 (Zhao, 2008)	188
2	Han JS, 2003, Trends Neurosci, V26, P17, DOI 10.1016/S0166-2236(02)00006-1 (Han, 2003)	178
3	Goldman N, 2010, Nat Neurosci, V13, P883, DOI 10.1038/NN.2562 (Goldman et al., 2010)	127
4	Han JS, 2004, Neurosci Lett, V361, P258, DOI 10.1016/J.NEULET.2003.12.019 (Han, 2004)	107
5	Ulett GA, 1998, Biol Psychiat, V44, P129, DOI 10.1016/S0006-3223(97)00394-6 (Ulett et al., 1998)	107
6	Zhang RX, 2014, Anesthesiology, V120, P482, DOI 10.1097/ALN.0000000000000101 (Zhang et al., 2014)	107
7	Melzack R, 1965, Science, V150, P971, DOI 10.1126/SCIENCE.150.3699.971 (Melzack and Wall, 1965)	100
8	Zimmermann M, 1983, Pain, V16, P109, DOI 10.1016/0304-3959(83)90201-4 (Zimmermann, 1983)	99
9	Ramsay DJ, 1998, JAMA-J AM MED ASSOC, V280, P1518, DOI 10.1001/jama.280.17.1518 (Ramsay, 1998)	92
10	Vickers AJ, 2012, Arch Intern Med, V172, P1444, DOI 10.1001/ARCHINTERNMED.2012.3654 (Vickers et al., 2012)	84

### 3.6 References with citation bursts

**Figure 9:**
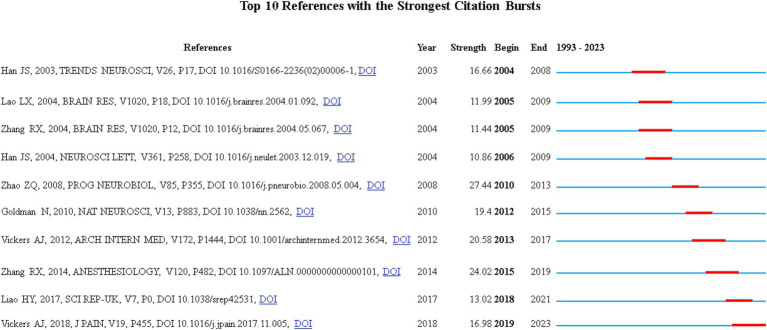
Top 10 references with strong citation bursts.

Citation-burst references are frequently cited by scholars in a particular field over a period of time. It indicates that the literature or the author’s research ideas have received special attention and researcher interest in the current time period and may represent a current research hotspot or trend. In our study, CiteSpace identified 10 references with strong citation bursts. In Figure 9, each bar represents a year, and the red bars represent strong citation bursts. The earliest citation bursts for the references appeared in 2004 and the latest in 2019. The reference with the strongest citation burst (intensity = 27.44) was titled “Neural mechanism underlying acupuncture analgesia” and was published by author Zhao Zhi-Qi in Progress In Neurobiology (Zhao, 2008), belonging to the citation burst from 2010 to 2013. The second strongest citation burst (intensity = 24.02) was the reference titled “Mechanisms of Acupuncture-Electroacupuncture on Persistent Pain” by Zhang et al. (2014) published in Anesthesiology, belonging to the citation bursts from 2015 to 2019. Overall, all 10 references had an outbreak intensity greater than 10 and a persistent intensity of 3 to 4 years.

### 3.7 Hotspots and Frontiers

**Figure 10:**
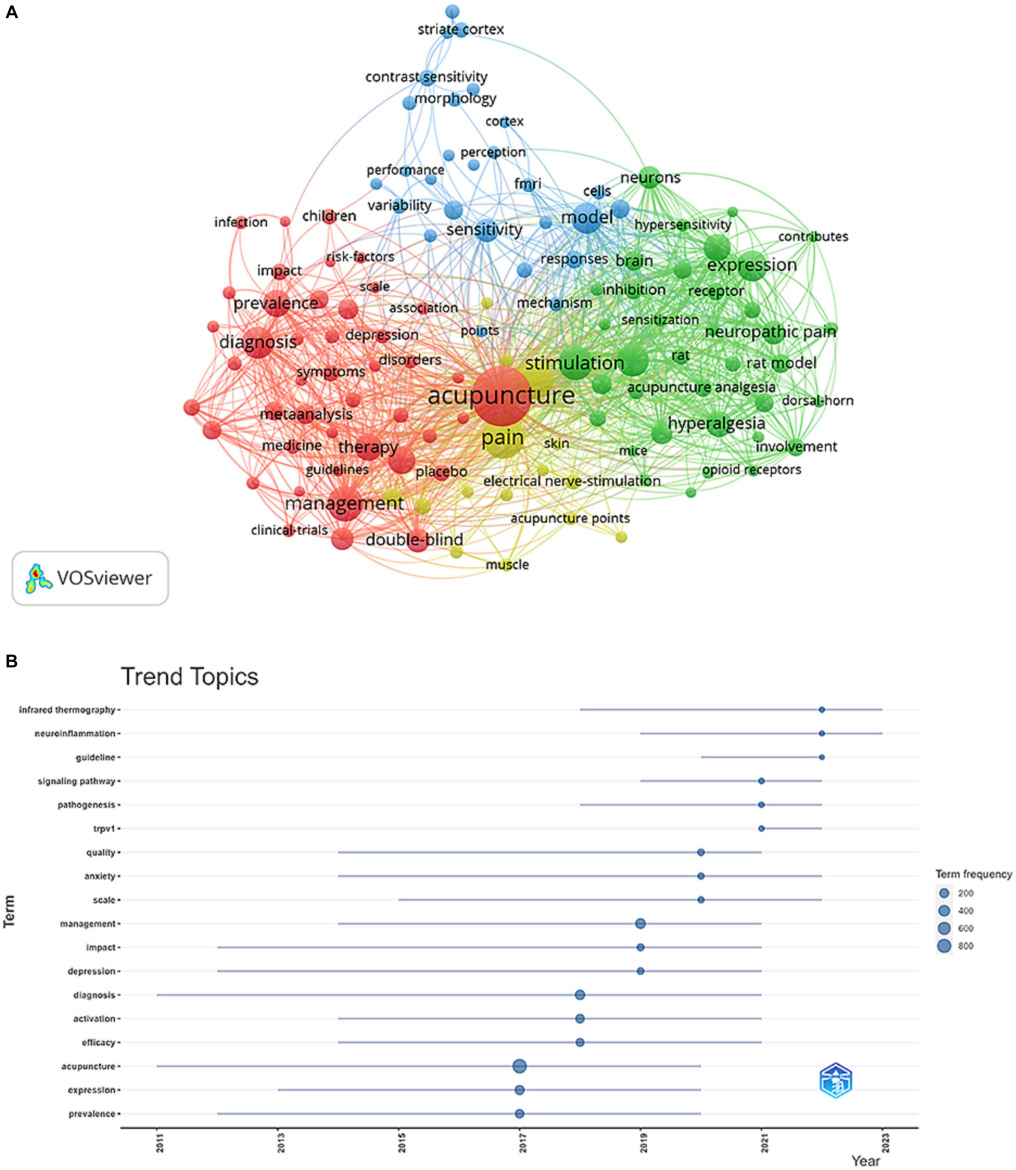
Keyword cluster analysis **(A)** and trend topic analysis **(B)** on research of acupoint sensitization and acupoint specificity (**A**: different nodes represent different keywords, the size of the nodes represents the frequency, and different colors represent different clusters; **B**: the size of the nodes represents the frequency).

Through the co-occurrence analysis of keywords, research hotspots in a certain field can quickly be captured. The top 20 high-frequency keywords in the field of acupoint sensitization and acupoint specificity research all had more than 100 times, representing the main directions in acupoint sensitization and acupoint specificity research (Table 6). Keywords with more than 30 occurrences were filtered and cluster analysis was performed using VOSviewer (Figure 10A). The thicker the lines between nodes, the stronger the connection between keywords. As shown in Figure 10A, a total of four clusters representing four research directions were obtained. Keywords in the red clusters included “complementary,” “alternative medicine,” “meta-analysis,” “randomized controlled trial,” “diagnosis, symptoms,” “management,” “prevalence,” “disorder,” “impact,” etc. Keywords in the green cluster included “stimulation,” “mechanism,” “sensitization,” “responses,” “expression,” “model,” “neuropathic pain,” “inflammation,” etc. Keywords in the blue cluster included “sensitivity,” “temperature,” “vision,” “points,” “model,” “performance,” “contrast sensitivity,” ‘FMRI,” etc. Finally, keywords in the yellow cluster included “acupuncture,” “pain,” “electroacupuncture,” “electrical nerve-stimulation,” “reliability,” etc.

**Table 6:** Top 20 keywords on research of acupoint sensitization and acupoint specificity.

Rank	Keywords	Counts	Rank	Keywords	Counts
1	Acupuncture	835	11	Model	220
2	Pain	426	12	Low-back-pain	168
3	Electroacupuncture	339	13	Therapy	170
4	Stimulation	276	14	Prevalence	167
5	Management	285	15	Neuropathic pain	127
6	Mechanisms	234	16	Analgesia	109
7	Expression	218	17	Efficacy	122
8	Diagnosis	247	18	Double-blind	138
9	Hyperalgesia	157	19	Neurons	116
10	Activation	170	20	Sensitivity	137

Trend theme analysis of keywords from 2017 onwards was conducted, and the results are shown in Figure 10B. From 2017 to today, the research direction has mainly focused on the temperature specificity of acupoints, the diagnosis of diseases sensitized by acupoints, and the study of the mechanism of acupoint sensitization. Major keywords included “infrared thermography,” “neuroinflammation,” “pathogenesis “infrared thermography,” “neuroinflammation,” “pathogenesis,” “TRPV1,” “signaling pathway,” “diagnosis,” of which “TRPV1,” “signaling pathway,” and “diagnosis” have high frequencies, and probably represent the current studies on acupoint sensitization and acupoint specificity.

## 4 Discussion

In this study, 4,940 publications on acupoint sensitization and acupoint specificity were obtained from the Web of Science database. The earliest publication was from 1981, and the number of publications per year from 1981 to 1990 was less than 10, thereby indicating that the related research was in its infancy. During 1991–2010, the number of publications per year increased year by year, with an average of 81 publications per year. During 2011-present, the number of publications began to increase substantially, with an average of 252 related publications per year, indicating that the research on acupoint sensitization and acupoint specificity has received increased attention and that the research is in an upward phase.

China and the United States are the primary countries performing research on acupoint sensitization and specificity, with China ranking first. Sixty percent of the top 10 research institutions are from China, and it was noted that there is more active collaboration between China, the USA, and United Kingdom. In terms of research institutions, there is a closer cooperation between Shanghai University of Traditional Chinese Medicine, Beijing University of Chinese Medicine, Chengdu University of Traditional Chinese Medicine, and the Chinese Academy of Chinese Medical Sciences. In addition, it was noted that some of the institutions have many publications but do not collaborate with others. This will not be conducive to the long-term development of academic research. Furthermore, China, the USA, and South Korea have high rankings in terms of publications, but the collaboration between them is limited. Therefore, it is strongly suggested that more in-depth collaboration between institutions in each country should be developed to jointly promote the progress of acupoint sensitization and acupoint specificity research.

Most studies on acupoint sensitization and acupoint specificity have been published in journals within broad categories of Complementary and Alternative Medicine. Among the top 15 journals in terms of the number of publications, journals with the highest impact factor are Neural Regeneration Research (IF = 6.1), American Journal of Chinese Medicine (IF = 5.7), and Frontiers in Neuroscience (IF = 4.3), thus indicating that the type of journal is the most popular journal in the field, and we can quickly track the latest results of related research in this type of journals. Most of the co-cited journals are Q1 and Q2 journals, which are high-quality international journals that provide reliable evidence to support the research on acupoint sensitization and acupoint specificity.

According to the authors, Wu Huangan, Fang Jianqiao, and Lin Yi-Wen published the most publications. Wu Huangan is from the Shanghai University of Traditional Chinese Medicine/Shanghai Institute of Acupuncture and Meridian Research, and his main research direction is the study of the basic principles and application of acupuncture. Fang Jianqiao is affiliated with the Zhejiang University of Traditional Chinese Medicine, and his main research interests are basic and clinical research on analgesic and immunomodulatory effects of acupuncture. Lin Yi-Wen is from the China Medical University Taiwan, and his main research interests are physiology/Acupuncture science. Overall, most of the authors are from the Chinese traditional medicine college and are engaged in acupuncture-related research.

According to the results of co-cited references, the 10 articles with the highest co-citation frequency were published more than 10 years ago, and these articles were co-cited by various other publications, suggesting that these articles serve as the basis of research in the field of acupoint sensitization and acupoint specificity research. In addition, from the references of the identified literature outbreaks, the main focus was on the following topics: the neural mechanism of acupuncture analgesia, the optimization scheme of electroacupuncture parameters, and the management of chronic pain by acupuncture. Co-occurrence analysis of keywords can help to quickly capture the distribution and evolution of research hotspots, and the results showed that the hotspots of acupuncture sensitization and acupoint specificity research hotspots were mainly focused on the study of the mechanism of acupuncture sensitization, the clinical application of acupuncture sensitization, and the observation of the biophysical characteristics of acupoints, which will need to be further explored in the future.

The analysis of keyword trends and topics in the last 5 years showed that “TRPV1,” “signaling pathway,” and “diagnosis” had a high frequency, thus indicating that Transient Receptor Potential Vanilloid 1 (TRPV1) may be involved in the process of acupoint sensitization (Bielefeldt and Davis, 2008). In addition, in several studies, it has been reported that the level of TRPV1 expression is higher in the acupoint area compared to the non-acupoint area (Abraham et al., 2011); the TRPV1 expression level is enhanced in the sensitized area compared to the non-sensitized area (Zhu, 2015); and TRPV1 knockout rats do not show thermal and pain sensitization after being harmfully stimulated by mustard oil, Freund adjuvant complete, etc., which suggests that the TRPV1 channel may be a key factor in the formation of thermal and pain hypersensitivity (Moriyama et al., 2005).

The theory of acupoints originated from “*Pain as acupoints” (以痛为输) i*n the *Miraculous Pivot (灵枢)*. From the historical process of the development and evolution of acupuncture points, it was observed that the Ashi point is the germination stage of the concept of acupoints, after which, the theory of acupoints evolved from the primary stage of having no clear location and no fixed names to the stage of having a fixed location and a specific name of acupoints, which are then attributed to the corresponding meridian acupoint system. Acupoint sensitization is closely related to the study of acupoint specificity. Under physiological conditions, acupoints are in a “silent” state, but when the target *Zang-Fu (脏腑)* is diseased, relevant areas of the body surface will experience sensory anomalies and changes in the biophysical properties of the “sensitized” state, i.e., the phenomenon of acupoint sensitization.

In this study, the existing literature on the phenomenon of acupoint sensitization was summarized as follows: (1) Internal pathological factors are a necessary prerequisite for the occurrence of acupoint sensitization, and among the many acupoint sensitization phenomena, a decrease in the sensory threshold of an acupoint is the most common manifestation of acupoint sensitization. Disease-related acupoints may exhibit increased sensitivity to pain and thermal stimulation, i.e., even slight stimulation can lead to enhanced sensation (Chae et al., 2007; Ben et al., 2012; Zhu, 2015). In previous studies, this pain and heat sensitization phenomenon has been observed in diseases, such as asthma (Lin et al., 2017), gastric ulcer (Fu et al., 2015), cervical spondylosis (Gao et al., 2015), and osteoarthritis (Jia et al., 2020) of the knee. However, the distribution of sensitized acupoints varies for different types of diseases, and most sensitized acupoints are located on meridians that are closely related to the disease. (2) In addition, changes in the biophysical characteristics of acupoints are one of the manifestations of the acupoint sensitization phenomenon. It has previously been reported that the thermal characteristics (temperature), electrical characteristics (electrical resistance), acoustic characteristics (acoustic wave frequency and amplitude), and optical characteristics (infrared spectra) of the acupoints, etc. were altered under different pathological states (Zhu, 2019b). For example, thermal characteristic studies of acupoints show increased skin temperature at some of the acupoints in primary dysmenorrhea (Fan et al., 2022), cholecystitis (Zhang et al., 2002), gastrointestinal dysfunction (Zheng et al., 2017), and facial paralysis (Mao et al., 2021). The results of electrical characteristics studies are inconsistent, with some studies showing that acupoints display high conductivity, i.e., low impedance (Ahn and Martinsen, 2007) and low resistance compared to non-acupoints (Reichmanis et al., 1975; Li et al., 2016). However, in other studies, decreased conductivity has also been reported (Wong, 2014) i.e., increased impedance (Ngai et al., 2011) and resistance (She et al., 2010) at acupoints associated with patients with asthma and dysmenorrhea. Therefore, the study of the electrical characteristics of acupoints needs to be further explored. Studies of acoustic and optical characteristics of acupoints have also observed a decrease in the frequency and amplitude of sound waves associated with acupoints in patients with diseases (Xia, 2019) and differences in the infrared spectrum (Zhou et al., 2012). (3) Moreover, some studies have shown that changes in the morphology of acupoints can be detected by traditional meridian diagnostic methods. For example, hardened subcutaneous tissue, such as nodules or streaks, is likely to be detected in some acupoint areas in disease states, or in other changes, such as pimples, depression, or color changes of the skin (Zhang et al., 2004).

**Figure 11:**
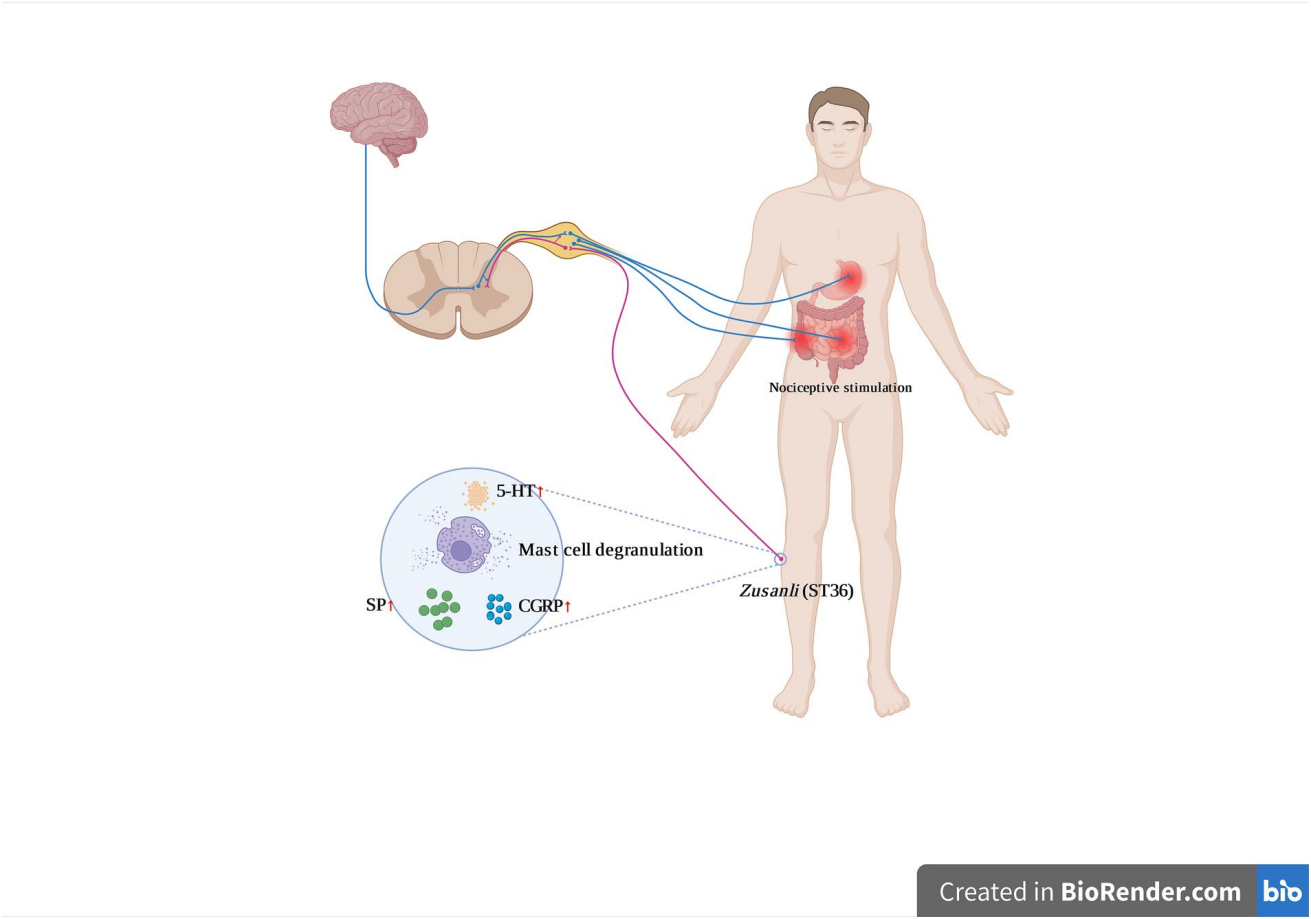
Possible mechanism of acupoint sensitization (this Figure created by www.biorander.com, 5-HT, serotonin; CGRP, calcitonin gene-related peptide; SP, P substance; 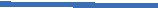 Upward conduction, 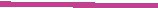 Downward conduction).

Regarding the mechanism of the occurrence of the acupoint sensitization phenomenon, existing studies agree (Fu et al., 2022) that the process of acupoint sensitization is dynamic, in which the process with the organs (internal organs or joint soft tissues), the spinal cord and supraspinal nervous system (central nervous system), and the acupoints (skin) are all involved in the process of acupoint sensitization. The injurious information generated by the damage to the organs (internal organs or soft tissues of joints) will be transmitted to the dorsal root ganglion, where the impulses are retrogradely transmitted to the periphery in the form of dorsal root reflexes through the activation of another adjacent dorsal root ganglion or retrogradely transmitted out to the periphery as an axonal reflex at the branch. The injurious stimulus that eventually travels to the periphery prompts nerve endings to release neuropeptides, such as substance P (SP) and calcitonin-gene related peptide (CGRP), which further stimulate the aggregation and degranulation of mast cells and the release of analgesic substances such as trypsin-like enzymes, histamine, and 5-HT, creating a local “inflammatory soup.” The formation of a local “inflammatory soup” of molecular pools leads to local sensitization of acupuncture points (Gold and Gebhart, 2010; Zhu, 2019b; Figure 11).

Many clinical studies have shown that better therapeutic effects can be achieved by needling pain-sensitizing acupoints than by needling traditional acupoints, and this phenomenon is particularly prominent in pain-based disorders (Itoh et al., 2004, 2007; Kim et al., 2017). These findings are supported by evidence-based medical evidence (Wong Lit Wan et al., 2015; Wang et al., 2016). To complete the evaluation of acupoint sensitization from different perspectives, different animal models, different clinical diseases, and different outcome indicators were used. The conclusions of the phenomenon of acupoint sensitization, although supportive, support that the evidence is heterogeneous. The results of these studies provide only preliminary evidence about the phenomenon and mechanisms of acupoint sensitization. However, with the current evidence, there are still some unresolved issues. First, the manifestations of acupoint sensitization are diverse, the criteria for sensitization have not yet been unified, and further in-depth research and determination of the criteria for defining acupoint sensitization are required. Second, randomized controlled trials for sensitized acupoints account for only a small fraction of all acupuncture clinical trials. Currently, mainstream studies still tend to be based on classical acupuncture theory, i.e., selecting acupoints based on meridian dialectics (Liang et al., 2009; Xing et al., 2013). Although the current evidence-based medical evidence on acupoint sensitization suggests that sensitized acupoints achieve better efficacy than traditional acupoints, the low quality of most clinical studies using sensitized acupoints reduces the quality of evidence-based medical evidence (Chen et al., 2012; Wang et al., 2016), Therefore, additional multicenter, large-sample clinical trials will need to be designed in the future to confirm the acknowledged superiority of the former approach. Third, most of the current basic studies on acupoint sensitization use an animal model of tail vein injection of Evans blue, after which the location of the sensitized acupoints is determined by the oozing point on the body surface. It is believed that acupoint sensitization is a neurogenic type of inflammatory response, and when there is Evans blue exudation on the body surface, it means that there is a local neurogenic inflammatory response, i.e., acupoint sensitization. However, there are various types of acupoint sensitization, and further research is needed to determine whether the use of Evans blue exudation is applicable to all types of acupoint sensitization. Fourth, the current mainstream view is that lowering the sensory threshold is the main performance characteristic of acupoint sensitization, but whether there is elevation of the sensory threshold (higher than normal), requires further in-depth studies. Finally, since acupoint sensitization is a dynamic process, current studies on the underlying mechanisms of acupoint sensitization have been reported in both the peripheral microcirculation and the central system. The detailed mechanisms involved, however, remain unclear. Therefore, it is necessary to identify reliable animal models of acupoint sensitization to further study the dynamic mechanism of acupoint sensitization.

Our study still has several limitations. First, due to the limitation of Citespace software and VOSviewer software, the data in this study were extracted from only one database, Web of Science, thus ignoring other data volume and some relevant articles may have been missed. Second, only studies published in English were included, which may mean that non-English-written publications were ignored, causing potential bias.

## 5 Conclusion

Acupoint sensitization and acupoint specificity studies have important research value and application prospects. Relevant publications have been increasing annually, indicating that researchers worldwide are becoming more interested in acupoint sensitization and specificity research. China and the USA have published most publications, but the cooperation and communication between countries and institutions need to be further strengthened. On the one hand, although the available evidence supports that needling sensitized acupoints achieve better clinical efficacy than traditional acupoints, the evidence is heterogeneous and needs to be supported by more high-quality clinical evidence. On the other hand, various types of acupoint sensitization have been reported so far, and given that the process of acupoint sensitization is dynamic, defining the criteria for defining acupoint sensitization and identifying reliable animal models of acupoint sensitization are necessary for further research on the dynamic mechanisms of acupoint sensitization.

## Data availability statement

The raw data supporting the conclusions of this article will be made available by the authors, without undue reservation.

## Author contributions

XL: Data curation, Formal analysis, Methodology, Resources, Validation, Writing – original draft, Writing – review & editing. YG: Data curation, Resources, Writing – original draft, Validation. DW: Data curation, Resources, Writing – original draft, Funding acquisition. JL: Data curation, Funding acquisition, Supervision, Writing – original draft. XF: Data curation, Formal analysis, Validation, Writing – original draft. HC: Supervision, Validation, Writing – original draft. GZ: Formal analysis, Methodology, Validation, Writing – original draft. HL: Visualization, Writing – original draft. XJZ: Visualization, Writing – original draft. XFZ: Visualization, Writing – original draft. JZ: Methodology, Software, Supervision, Visualization, Writing – review & editing, Conceptualization. YS: Writing – review & editing, Conceptualization, Funding acquisition, Methodology, Software, Supervision. XW: Visualization, Data curation, Formal analysis, Methodology, Resources, Validation, Writing – original draft, Writing – review & editing.

## Footnotes

^1^ https://www.bibliometrix.org
