# Corrigendum: Knowledge mapping of acupoint sensitization and acupoint specificity: a bibliometric analysis

**DOI:** 10.3389/fnins.2025.1640077

**Published:** 2025-06-17

**Authors:** Xuesong Wang, Xuxin Li, Yuanbo Gao, Di Wang, Jun Liu, Xisheng Fan, Hao Chen, Guang Zuo, Haiping Li, Xiaojun Zheng, Xifen Zhang, Juncha Zhang, Yanfen She

**Affiliations:** ^1^College of Acupuncture-Moxibustion and Massage, Hebei University of Chinese Medicine, Shijiazhuang, Hebei, China; ^2^Hebei International Joint Research Center for Dominant Diseases in Chinese Medicine and Acupuncture, Hebei University of Chinese Medicine, Shijiazhuang, Hebei, China

**Keywords:** bibliometrics, acupoint sensitization, acupoint specificity, CiteSpace, VOSviewer, R software

In the published article, there was an error in the Funding statement and some funding information was omitted. Original Funding statement:

The author(s) declare financial support was received for the research, authorship, and/or publication of this article. This study was supported by the National Key Research and Development Program of China (No. 2022YFC3500601), the National Natural Science Foundation of China (No. 81973755), and the Project on Hebei Administration of Traditional Chinese Medicine (Nos. 2022091 and 2021097).

The correct Funding statement appears below.

